# Studies on the serological markers for hepatitis B virus infection among type 2 diabetic patients

**DOI:** 10.1002/jcla.23464

**Published:** 2021-01-07

**Authors:** James A. Ndako, Obinna O. Nwankiti, Joy O. Olorundare, Stephen K.S. Ojo, Charles E. Okolie, Oludolapo Olatinsu, Victor T. Dojumo

**Affiliations:** ^1^ Department of Microbiology Landmark University Omu‐Aran Nigeria; ^2^ Department of Viral Research National Veterinary Research Institute Vom Nigeria; ^3^ Department of Microbiology Federal University Oye‐Ekiti Nigeria; ^4^ Department of Medical Laboratory Services Landmark University Medical Center Omu‐Aran Nigeria

**Keywords:** HBV, serological markers, type 2 diabetes mellitus

## Abstract

**Background:**

Hepatitis B infection is a public health concern globally. HBV can be associated with type II diabetes mellitus, as HBV outbreaks have been observed among diabetics in healthcare facilities. This study evaluates the prevalence of HBV infection among patients with type II diabetes mellitus.

**Method:**

A total of one hundred and eighty (180) diabetic patients and one‐hundred non‐diabetics (Controls) were recruited for this study. Structured questionnaires were administered to the consented participants to obtain relevant data. Sera samples obtained were screened using the HBsAg ELISA kit; CTK Biotech, Inc, while the 5 panel kit—rapid diagnostic test, was used to assay for serological markers. Questionnaires were used to obtain relevant information and demographic data.

**Result:**

Overall prevalence of HBV infection among diabetes patients was 13.3%. Breakdown showed 9 (5.0%) seropositivity was obtained among male subjects compared to 15(8.3%) recorded among the females, *P* = .834; *P* < .05. Subjects aged 41‐50 years recorded, 7(3.9%) positivity *P* = .774; *P* > .05. Educational status of participants showed 22 (12.2%) positivity among subjects with tertiary level of education *P* = .032; *P* < .05). Risk factors considered showed that 5(2.8%).seropositive subjects were alcoholic consumers (*P* value = .9711; *P* > .05). Result among non‐diabetics (Control) subjects showed (4%) seropositivity among the male subjects compared to (5.0%) seropositivity recorded among the female subjects (*P* = .739; *P* > .05).

**Conclusion:**

There is an indication of higher risk of HBV infection among type 2 diabetic patients when compared to non‐diabetics. There is the need for more research on this area of study, to further validate the association between HBV infection and Diabetes Mellitus.

## Graphical abstract

Overall prevalence of HBV infection among diabetes patients was 13.3%. Breakdown showed 9 (5.0%) seropositivity was obtained among male subjects compared to 15 (8.3%) recorded among the females. Subjects aged 41‐50 years recorded a higher positivity of 7 (3.9%). Risk factors considered showed that 5(2.8%) of the seropositive subjects were alcoholic consumers. There is an indication of higher risk of HBV infection among type 2 diabetic patients when compared to non‐diabetics. General surveillance through routine screening of diabetics in endemic regions with HBV infection is highly advocated to identify those with the hepatitis B infection. Establishing appropriate measures to manage the infection such as vaccination and public health enlightenment to the populace on the scourge of this infectious agent is strongly advocated. Improved diagnosis for early detection of HBV infection among diabetic patients to reduce further complications is strongly recommended. There is the need for more research to further validate the association between HBV infection and diabetes mellitus.

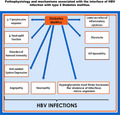

**Abbreviations**
5PTK: five panel test kit
DM: diabetes Mellitus
ELISA: enzyme‐Linked Immunosorbent Assay
*HBcAb*: hepatitis B core antibodies
HBeAb, hepatitis B e‐ antibody
*HBeAg*: hepatitis B e‐ antigen
*HBsAb*: hepatitis B surface Antibody
*HBsAg*: hepatitis B surface Antigen
HBV: hepatitis B Virus
T2DM: Type 2 Diabetes Mellitus

## 1 BACKGROUND

Hepatitis B virus (HBV) is one of the most common chronic infections worldwide and the leading cause for hepatocellular carcinoma (HCC) worldwide.^1^ Based on WHO report, one‐third of the world population estimated at about 2 billion individuals are HBV‐infected, including studies showing that about 240 million individuals are chronic carriers.^2^ Transmission of this virus occurs via exchange of bodily fluid such as blood, semen, and perinatal transmission. Hepatitis B has been majorly associated with hepatocellular carcinoma, cirrhosis, and chronic hepatitis, and this makes it identifiable as a major health issue in various developing countries.^3^ An increased rate of this disease occurrence has been recorded in regions like East Asia and sub‐Saharan Africa where 5%‐10% of adults in these locations come up with chronic hepatitis infection as compared to other areas.^4^

Hepatitis virus is a member of the Hepadnaviridae family, possessing a small, enveloped DNA virus called the “Australia antigen” and later referred to as hepatitis B virus. Studies have shown that HBV has contributed to the increased morbidity rate worldwide. This virus is known to cause about 50% of cases of hepatocellular carcinoma and 30% of cases of cirrhosis globally.^5^, ^6^ Further studies showed that the hepatitis B surface antigen (HBsAg) was known to be present in patient's serum.^7^ This surface antigen is present on the outer envelope alongside an inner nucleocapsid consisting of hepatitis B core antigen (HBcAg).^8^

Diagnosis of this virus is done by blood screening for the surface antigen. To enable most precise diagnosis and to determine the infectious rate, the diagnosis and follow‐up of chronic hepatitis B virus (HBV) infection relies on laboratory viral biomarkers. There are two major categories of HBV biomarkers. The first one is serology, a term comprising the detection and quantification of viral antigens and viral specific antibodies, and the second is nucleic acid testing (NAT) for the detection and quantification of HBV genome and its RNA transcripts.^9^ The spread of this virus is rapidly on the increase globally; as large numbers of individuals are unaware of the ravaging effect of this virus nether do they know their status mostly in identified endemic regions. Complications can occur after prolonged infection with this virus and due to its asymptomatic nature. Transmission of the virus can easily occur without adequate precaution..^10^

Based on the increasing prevalence of HBV, various studies have been carried out on this virus with less information on its association with diabetic patients. HBV outbreaks have been observed among diabetics in healthcare facilities and nursing homes. Diabetic patients are vulnerable to hepatotropic infections as a result of recurring hospitalization and blood screening.^11^ Studies carried out by the National Health and Nutrition Examination Survey revealed an increased prevalence of hepatitis B among diabetic patients as compared to non‐diabetic patients. Diabetic patients, due to their defective levels of T lymphocytes are vulnerable to viral infections as a result of an immune compromised system.

## 2 METHOD

### 2.1 Study area and population

The study was conducted at the Federal Teaching Hospital Ido‐Ekiti which is a tertiary health institution. The study population comprised of randomly selected confirmed diabetic patients attending the outpatient department of the Teaching Hospital.

### 2.2 Ethical permit and consent

A proposal of the project was submitted to the Ethical Review Committee of the Federal Teaching Hospital Ido‐Ekiti, where ethical permit was sought for and obtained with protocol number: ERC/2018/02/27/103B.

### 2.3 Inclusion and exclusion criteria

Individuals confirmed for type 2 diabetes mellitus diabetes were recruited for the study. Persons who showed no interest in the study and are not diabetic were excluded from the study.

### 2.4 Questionnaire and sample size

The sample size for research work was obtained from one‐hundred and eighty (180) volunteers, and the recruits were properly informed about the study and their consents obtained. Well‐structured questionnaires were used to collect demographic data and other pertinent information.

### 2.5 Sample collection and processing

Aseptic collection of three to five (3‐5) mL of blood from each diabetic patient was done. At room temperature, blood samples were left undisturbed to allow clotting. Thereafter, sera obtained were dispensed into a clean, dry cryovial and stored at −20°C prior use. Screening of the sera was done for antibodies to HBV with the use of ELISA kits (Fortress Diagnostic Limited), while the 5 panel kit—rapid diagnostic test, was used to assay for serological markers. Standard procedures were strictly adhered to during the assaying process.

### 2.6 Sample processing

Sera samples obtained were screened for HCV antibodies using ELISA technique according to the manufactures manual. The 5 panel kit—rapid diagnostic test, was used to assay for serological markers serum ALT was also assayed for, according to manufacturer's manual. Hepatitis B ELISA and ALT kits were stored in the refrigerator at 4°C prior to use. Sera samples were analyzed at Landmark University Medical Laboratory.

### 2.7 Data analysis

Filled questionnaires were crosschecked manually for correct entry of data. The data were analyzed using the SPSS software package, and chi‐square test was used to compare several variables while the critical level for statistical significance was set at *P* = 5% (.05).

## 3 RESULTS

The total sample comprises of 109 (60.6%) female samples and 71 (39.4%) male samples. The distribution in age among individuals analyzed is 0‐100 years. A total of 24 (13.3%) samples tested positive for HBV among the 180 diabetic patients tested while 156 (86.7%) tested negative, and this result gives HBV a prevalence of 13.3%.

One‐hundred (100) non‐diabetics (Controls) subjects screened comprise of 48 males and 52 female, with a record of 4% positivity for the male control subjects as compared to 5% positivity recorded among the female subjects, *P* = .739; *P* > .05.

From the 24 positive samples, 9 (5.0%) males tested positive compared to 15 (8.3%) positivity among the female diabetic subjects screened. *P* = .834 (*P* > .05) indicating statistical insignificance otherwise hypothesis not significant, showing that HBV infection among diabetic patients is not dependent on the gender (Tables 1 and 2).

**Table 1:** Distribution of HBV among diabetics based on gender

Sex	Total Number of Respondents examined (%)	Number of Positive Respondents (%)	Number of Negative Respondents (%)
Male	71 (39.4%)	9 (5.0%)	62 (34.4%)
Female	109 (60.6%)	15 (8.3%)	94 (52.2%)
Total	180 (100%)	24 (13.3%)	156 (86.6%)

Note

*P* = .834; *P* < .05.

**Table 2:** Distribution of HBV among B non‐diabetics (Control Subjects) based on gender

SEX	Number tested	HBV	
		Positive %	Negative%
Male	48	4 (4%)	44 (44%)
Female	52	5 (5%)	47 (47%)
Total	100	9 (9%)	91 (91%)

Note

*P* = .739; *P* > .05.

The age group of 0‐20, 20 individuals where tested with a total of 19(95%) testing negative and 1(5.0%) testing positive in this age group. Between 21 and 30 years, 20 (11.1%) individuals were tested: 17 (85.0%) tested negative while 3 (15.0%) tested positive. In the age group between 31 and 40 years, a total of 37 (20.6%) were tested and 32 (86.5%) were negative while 5 (13.5%) were positive. Between age group 41 and 50, a total of 40 (22.2%) were tested, having 7 (17.5%) individuals positive and 33 (82.5%) negative. In the age range 51‐60, 24 (13.3%) individuals were tested and 22 (991.7%) were negative while 2 (8.3%) were positive. Individuals above the 60 years of age a total of 39 (21.7%) persons were tested, having 6 (15.4%) positive and 33 (84.6%) negative (Tables 3 and 5).

**Table 3:** Distribution of HBV among diabetics based on Age

Age Category	Total Number of Respondents examined (%)	Number of Positive Respondents (%)	Number of Negative Respondents (%)
Below 20	20 (11.1%)	1 (0.6%)	19 (10.6%)
21‐30	20 (11.1%)	3 (1.7%)	17 (9.4%)
31‐40	37 (20.6%)	5 (2.8%)	32 (17.8%)
41‐50	40 (22.2%)	7 (3.9%)	33 (18.3%)
51‐60	24 (13.3%)	2 (1.1%)	22 (12.2%)
Above 60	39 (21.7%)	6 (3.3%)	33 (18.3%)
Total	180 (100%)	24 (13.4%)	156 (86.6%)

Note

*P* = .774; *P* > .05.

On the basis of the marital status, 130 (72.2%) married subjects were screened out of which 22 (12.2%) tested positive while 108 (60.0%) tested negative. Among the 41 (22.8%) single subjects screened, 2 (1.1%) tested positive while 39(21.7%) were negative (Table 4).

**Table 4:** Distribution of HBV among diabetics Based on Marital status

Marital Status	Number of Positive Respondents (%)	Number of Negative Respondents (%)	Total Number of Respondents (%)
Single	2 (1.1%)	39 (21.7%)	41 (22.8%)
Married	22 (12.2%)	108 (60.0%)	130 (72.2%)
Divorced	0 (0.0%)	9 (5.0%)	9 (5.0%)
Total	24 (13.3%)	156 (86.7%)	180 (100%)

Note

*P* = .068; *P* < .05.

The result based on educational status of subjects screened, the prevalence of 2 (1.1%) was recorded among subjects with a minimum of primary educational status. The prevalence of 3 (1.7%) was recorded among those with secondary level of education, while 18 (10.0%) positivity was recorded among individuals with tertiary level of education (Table 5).

**Table 5:** Distribution of HBV among diabetics Based on Educational qualification

Qualification	Total Number of Respondents examined (%)	Number of Positive Respondents (%)	Number of Positive Respondents (%)
Primary	6 (3.3%)	2 (1.1%)	4 (2.2%)
Secondary	25 (13.9%)	3 (1.7%)	22 (12.2%)
Tertiary	148 (82.2%)	18 (10.0%)	130 (72.2%)
No Education	1 (0.5%)	1 (0.5%)	0 (0.0%)
Total	180 (100%)	24 (13.3%)	156 (86.7%)

Note

*P* = .032; *P* < .05.

Based on the demographic factors, a prevalence of 9 (5.0%) was recorded among subjects engaging in trading, while 11 (6.1%) prevalence rate was recorded among civil workers, 3 (1.7%) prevalence among industrial workers, and 1 (0.6%) prevalence was recorded among subjects who were students, *P* = value of .240; *P* > .05 (Table 6).

**Table 6:** Distribution of HBV among diabetics Based on Occupational distribution of subjects screened

Occupation	Total Number of Respondents (%)	Number of Positive Respondents (%)	Number of Negative Respondents (%)
Trading	64 (35.6%)	9 (5.0%)	55 (30.6%)
Civil Servant	64 (35.5%)	11 (6.1%)	53 (29.4%)
Industry	18 (10.0%)	3 (1.7%)	15 (8.3%)
Student	34 (18.9%)	1 (0.6%)	33 (18.3%)
Total	180 (100%)	24 (13.4%)	156 (86.6%)

Note

*P* = .240; *P* > .05.

On the basis of risk factors among subjects screened, 2.8% prevalence was recorded among alcohol consumers, while 1.7% prevalence was recorded among individuals with history of tribal mark/ tattoos. 1.7% positivity was recorded among individuals with history of sharing sharp objects (Table 7).

**Table 7:** Distribution of HBV among diabetics based on Risk factors examined

Risk Factor	Response	Number of Positive Respondents	Number of Negative Respondents	Total Number of Respondents Examined	Chi‐Square
Alcohol Consumption	Yes	5 (2.8%)	32 (17.8%)	37 (20.6%)	0.001
	No	19 (10.5%)	124 (68.9%)	143 (79.4%)	*df* = 1
	Total	24 (13.3%)	156 (86.7%)	180 (100.0%)	*P* value = .9711
Tribal Marks and Tattoos	Yes	3 (1.7%)	16 (8.9%)	19 (10.6%)	0.111
	No	21 (11.6%)	140 (77.8%)	161 (89.4%)	*df* = 1
	Total	24 (13.3%)	156 (86.7%)	180 (100.0%)	*P* value = .7391
Engage in Risky Behaviors	Yes	1 (0.6%)	21 (11.6%)	22 (12.2%)	1.675
	No	23 (12.8%)	135 (75.0%)	158 (87.8)	*df* = 1
	Total	24 (13.3%)	156 (86.7%)	180 (100.0%)	*P* value = .1956
Sharing of Razor Blade/Scissors/Nail cutter	Yes	3 (1.7%)	53 (29.4%)	56 (31.1%)	4.475
	No	21 (11.6%)	103 (57.3%)	124 (68.9%)	*df* = 1
	Total	24 (13.3%)	156 (86.7%)	180 (100.0%)	*P* value = .0344
Non‐spousal Sexual Intercourse in 3‐5 y	Yes	1 (0.6%)	6 (3.3%)	7 (3.9%)	0.006
	No	23 (12.8%)	150 (83.3%)	173 (96.1%)	1
	Total	24 (13.4%)	156 (86.6%)	180 (100.0%)	*P* value = .9397

Based on clinical history of subjects screened, 2.8% prevalence was recorded among individuals with family history of HBV infection, compared to 2.2% prevalence was recorded among care givers (Table 8).

**Table 8:** Distribution of HBV among diabetics based on Clinical History

Medical History	Response	Total Number of Respondents Examined (%)	Number of Positive Respondents (%)	Number of Negative Respondents (%)	Chi‐Square
Hepatitis by Family Members	Yes	32 (17.8%)	5 (2.8%)	27 (15.0%)	0.177
	No	148 (82.2%)	19 (10.5%)	129 (71.7%)	*df* = 1
	Total	180 (100.0%)	24 (13.3%)	156 (86.7%)	*P* value = .6741
Previous Record of Hepatitis Virus	Yes	7 (3.9%)	2 (1.1%)	5 (2.8%)	0.000
	No	173 (96.1%)	22 (12.2%)	151 (83.9%)	*df* = 1
	Total	180 (100.0%)	24 (13.3%)	156 (86.7%)	*P* value = 1.000
Received Blood Transfusion in Time Past	Yes	13 (7.2%)	0 (0.0%)	13 (7.2%)	0.791
	No	167 (92.7%)	24 (13.3%)	143 (79.4%)	1
	Total	180 (100.0%)	24 (13.3%)	156 (86.6%)	*P* value = .3737
Blood Donation	Yes	13 (7.2%)	0 (0.0%)	13 (7.2%)	2.156
	No	167 (92.7%)	24 (13.3%)	143 (79.4%)	*df* = 1
	Total	180 (100.0%)	24 (13.3%)	156 (86.6%)	*P* value = .1420
Seen/Care for an Hepatitis B or C patient	Yes	38 (21.1%)	4 (2.2%)	34 (18.9%)	0.328
	No	142 (78.9%)	20 (11.1%)	122 (67.8%)	*df* = 1
	Total	180 (100.0%)	24 (13.3%)	156 (86.7%)	*P* value = .5666

The assay carried out on the positive samples, using the 5‐panel kit, showed that 37.5% positivity was recorded among individuals with HBsAg, and 8.3% was recorded for subjects with HBsAb. However, 4.2% positivity was recorded for subjects with HBeAg, 45.8% positivity was noted for subjects with HBeAb, and 54.2% positivity was recorded among subjects with HBcAb. (Table 9).

**Table 9:** Overall prevalence of HBV markers among seropositive subjects

S/N	AGE	SEX	OD value	HBsAg	HbsAb	HbeAg	HbeAb	HBcAb
1	42	F	1.03	Negative	Negative	Negative	Negative	Negative
2	52	M	16.92	Positive	Negative	Negative	Positive	Positive
3	75	M	1.45	Negative	Negative	Negative	Negative	Negative
4	59	F	1.29	Negative	Negative	Negative	Negative	Negative
5	34	F	6.41	Positive	Negative	Negative	Weakly Positive	Positive
6	76	F	1.10	Negative	Negative	Negative	Negative	Negative
7	68	F	5.91	Negative	Negative	Negative	Negative	Positive
8	62	F	5.64	Negative	Negative	Positive	Negative	Negative
9	30	F	17.02	Positive	Negative	Negative	Positive	Positive
10	40	F	8.68	Positive	Negative	Negative	Positive	Positive
11	38	F	1.15	Negative	Positive	Negative	Negative	Negative
12	48	M	1.15	Negative	Negative	Negative	Negative	Positive
13	45	M	1.03	Negative	Negative	Negative	Negative	Negative
14	30	F	26.67	Positive	Negative	Negative	Positive	Positive
15	30	F	14.88	Positive	Negative	Negative	Positive	Positive
16	101	F	26.67	Positive	Negative	Negative	Positive	Positive
17	41	M	1.09	Negative	Negative	Negative	Negative	Negative
18	50	M	1.06	Negative	Negative	Negative	Negative	Positive
19	67	M	1.14	Negative	Weakly Positive	Negative	Negative	Positive
20	41	F	1.13	Negative	Negative	Negative	Negative	Negative
21	40	M	16.93	Positive	Negative	Negative	Positive	Positive
22	19	F	2.03	Negative	Negative	Negative	Positive	Negative
23	44	F	1.17	Negative	Negative	Negative	Positive	Negative
24	35	M	13.7	Positive	Negative	Negative	Positive	Positive

Abbreviations: F, Female; M, Male; OD value, optical density value.

## 4 DISCUSSION

HBsAg positivity was more prevalent among diabetic patients (13.3%), compared to the non‐diabetic subjects (9%). Although this difference was statistically insignificant, it was of clinical importance. The result obtained from the sera samples screened among diabetic patients showed a prevalence rate of 13.3%.This result is comparable to the study carried out at Ahmadu Bello University, Zaria among diabetes where HBV prevalence of 12.5% was recorded.^12^ Our findings also correspond with a national survey carried out in Nigeria where the prevalence of 12.2% was recorded.^13^ A study done in Taiwan also showed a related prevalence of 13.5% among diabetic patients.^14^ However, the result obtained from this research was higher in comparison with a study carried out in China and Turkey which recorded 3.8% and 5.1% prevalence among diabetic patients, respectively.^15^, ^16^

The distribution of HBV among male and female gender recorded 5.0% and 8.3% prevalence, respectively, statistical analysis carried out showed that HBV infection is not dependent on gender of subjects screened. This differences may be attributed to the larger number of female subjects recruited for this study, and similar studies suggest that both gender are equally susceptible to HBV infection.^17^ Hence, it can be concluded that the distribution of HBV based on gender is not significant. Although a study carried out by Joanah et al (2016) also attests to an increased occurrence of HBV among females by a relative rate of 1.0%, Donbraye et al (2014) also recorded a higher prevalence of HBV in females compared to males in a study carried out in Osun State.^18^, ^19^

Considering age of subjects screened, a higher prevalence of 3.9% was recorded among subjects aged 41‐50 years compared to the other age groups. The result obtained in this study is in contrast to a similar study in turn contradicts the outcome of studies conducted by Mohd et al (2013), which reported a higher prevalence among subjects aged 25‐34 years.^20^ A similar study conducted by Malewe et al (2017) at Lome showed a higher prevalence rate of 21.67% among individuals within the age range of 30‐39. However, results obtained in this study among the various age groups screened does not show any statistically significant = 0.774; *P* > .05 (Table 3).

Marital status has been observed to be a major determining factor in the occurrence of HBV infection. Studies conducted by Abdollahi et al (2006) showed a higher prevalence among unmarried subjects compared to those married.^21^ Interestingly, this work showed a higher prevalence of 12.2% among the married subjects compared to subjects of other marital status. These differences can be attributed to probable cultural diversities in such communities. McQuillan et al (1998) also recorded a significant incidence rate among widows than individuals of other marital status^22^, ^23^ (Table 4). This could be as a result of the sexual promiscuity of any of the partners. The rate of transmission of this virus among diabetic sexual partners can be reduced via the introduction of HBV vaccines, use of contraceptives, and public enlightenment campaign to educate the populace on preventive measures against the HBV infection.

The result based on educational status of subjects screened the prevalence of 2 (1.1%) was recorded among subjects with a minimum of primary educational status. The prevalence of 3 (1.7%) was recorded among those with secondary level of education, while 18 (10.0%) positivity was recorded among individuals with tertiary level of education (Table 5). The difference in prevalence rate based on the educational status of subjects studied was, however, statistically significant. The importance of HBV vaccination in diabetes patients is not much disseminated, although public health policies recommend vaccination through educational campaigns to patients and public health professionals.

Based on the demographic factors, a prevalence of 9 (5.0%) was recorded among subjects who engaged in trading, while 11 (6.1%) prevalence rate was recorded among civil workers, 3 (1.7%) prevalence among industrial workers, and 1 (0.6%) prevalence was recorded among subjects who were students,.(*P* = value: .240; *P* > .05), (Table 6). Though not statistically significant, risk factor studied shows a high prevalence among subjects with history of unsafe social practices, with a prevalence of 5 (2.8%) among alcohol consumers. This result is similar to the study of Tekin et al (2015) showing an HBV incidence rate of 16.3% among alcohol consumers employed for a study in Turkey.^24^ Based on the clinical history of infected individuals, 1.1% prevalence rate was recorded among individuals with family history of hepatitis infection. The susceptibility of such individuals may be linked to a hereditary factor in existence.^25^ The association between HBV infection and DM is related to the direct effects of HBV on pancreatic lymphocytes, resulting in decreased insulin production, while patients with HBV‐protein X reduce the expression of insulin receptor proteins among such subjects.^14^

Considering the seromarkers for HBV infection from this study, the highest rate of positivity recorded involved the HBcAb with 54.2% among the diabetic subjects screened. According to Rehermann,^26^ patients with resolved infection have persistence of anti‐HBc for life, although the total anti‐HBc is predominantly consisting of IgG at about four to 6 months following the appearance of anti‐HBc. However, hepatitis B core antibody (anti‐HBc) is the preferable serologic test to establish a history of previous infection. Anti‐HBc is the earliest antibody to develop in response to HBV infection, appearing as IgM anti‐HBc. Anti‐HBc typically persists for life as IgG anti‐HBc after 6 months of the infection. Thus, IgM anti‐HBc is a marker of acute infection while IgG‐anti HBc is a marker of past infection. Isolated anti‐HBc refers to the presence of IgG anti‐HBc in the absence of HBsAg and anti‐HBs.^27^

Anti‐HBe positivity showed a record of 45.8%, in the natural history of HBV infection, the most important event is HBeAg seroconversion characterized by loss of HBeAg and development of antibody to HBeAg (anti‐HBe). This generally occurs years after replicative phase and indicates a transition to a low/non‐replicative phase with a potential for resolution of infection and improvement of necro‐inflammation in the liver.^28^

The anti‐HBs recorded 8.3% positivity. According to Lee,^29^ chronic infection is indicated in the occurrence of HBsAg for a period of 6 months or more after an acute infection. Chronic infection is also indicated in the absence of IgM anti‐HBc and presence of HBsAg in a single serum sample. Patients who have been vaccinated or those who recovered from HBV infection have protective immunity as a result of antibody production against HBsAg (anti‐HBs).^30^

Anti‐HBe recorded 4.2% among the screened subjects positive for HBV; previous study reported the presence of HBeAg in serum to indicate active viral replication.^30^ The continued presence of HBeAg generally reflects higher HBV DNA levels and greater infectiousness. Some patients with chronic HBV infection may have resolution of their HBeAg alongside the appearance of anti‐HBe, which correlates with low (or absent) HBV levels and relatively normal levels of hepatic aminotransferase levels.^29^ Zhang et al^30^ in his studies recorded 1.6% HBeAg positivity among young adults, while the rate of positivity decreased with age due to the spontaneous seroconversion to the antibody against the HBeAg (Anti‐HBe).

Anti‐HBs which indicate antibody to the HBsAg showed 35 (17.5%) positivity among children, 20.0% among young adults, 11.6% among pregnant women, 5.0% among blood donors, and 14.5% among apparently healthy subjects. While anti‐HBs recorded 18.7% positivity among subjects aged 15‐19 years followed by 11.7% positivity among subjects aged 5‐9 years, subjects aged 25‐29 years recorded 8.4% positivity to the anti‐HBs. For those patients who resolve their infection, HBsAg disappears at about 3‐6 months, often just prior to the detection of antibodies to hepatitis B surface antigen (anti‐HBs).

The presence of anti‐HBs following acute infection generally indicates recovery and protective\immunity against re‐infection. In addition, patients with resolution of infection have disappearance of HBeAg and development of antibodies to hepatitis B e antigen (anti‐HBe). Patients with resolved infection have persistence of anti‐HBc for life, but about 4‐6 months after the appearance of anti‐HBc, the total anti‐HBc predominantly consists of IgG. Some patients with self‐limited infection, however, may still have low levels of HBV DNA in blood; whether the HBV DNA is part of intact virions remains unknown (Yang et al,^31^).

Earlier studies (Zhang et al,^30^) reported that improvement in vaccination strategies could be attributed to upsurge in HBsAb positivity, which should increase the confidence of the population on the immunization process as a safe guard against HBV infection.

## 5 CONCLUSION

In conclusion, this study found that the risk of HBV infection after the onset of T2DM recorded no significance statistical difference compared to risk factors among patients without the T2DM. However, individuals infected with T2DM had a higher incidence rate of HBV in all the age ranges studied when compared to non‐diabetic patients. General surveillance through routine screening of diabetics in endemic regions with HBV infection is highly advocated, to identify those with the hepatitis B infection.

Establishing appropriate measures to manage the infection such as vaccination and public health education to enlighten the populace on the scourge of this infectious agent coupled with improved diagnosis for early detection of HBV infection among diabetic patients is strongly recommended, for policy making and implementation in such communities. This is to reduce to the barest minimum further complications among similar subjects in the population.

## Data Availability

The datasets used and/or analyzed during the current study are available from the corresponding author on reasonable request.
